# High-Temperature Intrinsic Defect Chemistry of Li_8_PbO_6_ Ceramic Breeding Material

**DOI:** 10.1021/acs.jpcc.3c04186

**Published:** 2023-11-02

**Authors:** Andrew W. Davies, William D. Neilson, Reece T. Bedford, Samuel T. Murphy

**Affiliations:** † Department of Engineering, Lancaster University, Bailrigg, Lancaster LA1 4YW, U.K.; ‡ Los Alamos National Laboratory, Los Alamos, New Mexico 87545, United States

## Abstract

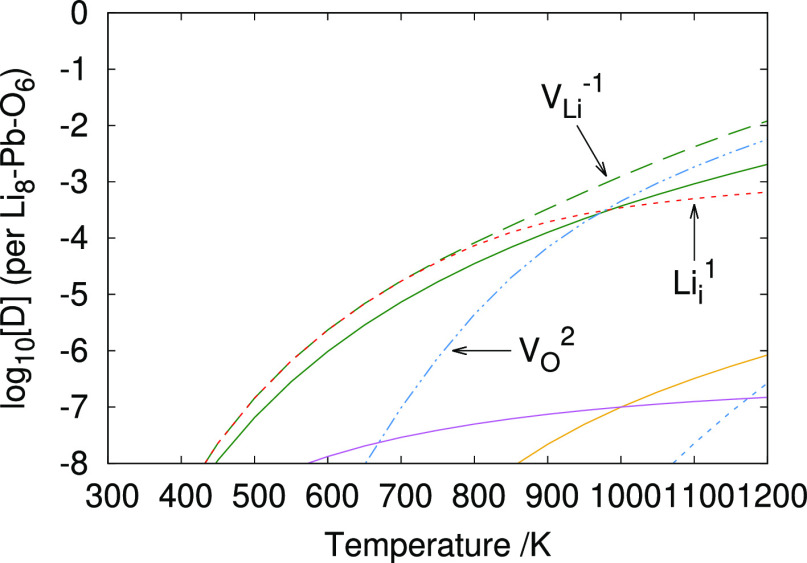

Understanding the intrinsic defect chemistry of tritium breeder materials proposed for use in future fusion reactors is imperative, as certain defects may act as traps leading to retention of tritium in the ceramic matrix. In this paper, we use combined density functional theory simulations with simple thermodynamics to explore the intrinsic defect chemistry of octalithium plumbate (Li_8_PbO_6_) as a function of both temperature and oxygen partial pressure. Importantly, we consider vibrational contributions to the energies of the reference states used in the calculations of the defect formation energies. Our results indicate that including these temperature effects can modify the predicted defect chemistry for materials at a high temperature. For Li_8_PbO_6_, the defect chemistry is predicted to be dominated by the V_Li_^–1^ defect, which will likely act as a trap for tritium. The charge compensating mechanism is predicted to change as a function of the conditions, with the Li_*i*_^+1^ interstitial defect providing compensation at low temperatures and the V_O_^2+^ vacancy defect occurring close to the Li_2_O saturation limit.

## Introduction

An essential criterion for the development of a fleet of future fusion reactors is finding a means of generating sufficient tritium to maintain self-sufficiency. Due to the short half-life of tritium of 12.32 years,^1^ tritium exists in only trace quantities in seawater.^2^ In fact, naturally occurring tritium is insufficient to sustain even a single tokamak fusion reactor. Therefore, it is imperative that a method for generating tritium sustainably is developed.

Currently, the proposed method for tritium generation is to exploit the high-energy neutron produced by the D–T fusion reaction (eq 1) to drive the transmutation of lithium via eqs 2 and 3
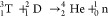
1
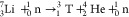
2
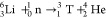
3

There are a number of blanket concepts being developed for future fusion reactors. These are typically classified according to the phase of the breeding material itself. Solid breeder materials are typically ceramic oxides in pebble form in concepts, such as the EU’s Helium Cooled Pebble Bed blanket.^3^ It is crucial that the ratio of tritium recovered from the blanket relative to the amount used in the plasma is greater than 1 (i.e., tritium breeding ratio, TBR > 1). To account for losses, due to radioactive decay and seepage into reactor components, it is expected that the TBR must be at least 1.1.^4^ To ensure this, a neutron multiplier is introduced to increase the number of neutrons available to drive the transmutation of lithium into tritium.

At present, the two primary candidate ceramic breeder blanket materials for the ITER and DEMO projects are the ceramics lithium metatitanate^5^ (Li_2_TiO_3_) and lithium orthosilicate^6^ (Li_4_SiO_4_). Developing blanket designs that deliver the requisite TBR using these ceramics requires the use of beryllium as an external neutron multiplier. This is problematic as beryllium is known to contain trace impurities of species, such as uranium, which when irradiated, cause production of minor actinides and increase the total volume of long-term radioactive waste.^7^ Because of this, it is imperative to explore alternative blanket materials that can achieve the desired TBR without relying on the use of Be as a multiplier.

A review article composed by Hernandez and Pereslavtsev^8^ suggested that octalithium ceramics might offer higher TBRs compared with other ceramic materials; however, stability issues are expected to occur at high temperatures for these lithium-rich materials. Our previous work examined the thermodynamic stability of a range of different octalithium ceramics within the quasi-harmonic approximation (QHA) and predicted that of the candidates tested, octalithium plumbate (Li_8_PbO_6_, with a predicted TBR of 1.21), appears thermodynamically stable enough to justify further investigation.^27^ The work of Hayashi et al. showed that Li_8_PbO_6_ exhibits excellent tritium release characteristics;^9^ however, what is less clear is how this will change during the operation. Lithium burn-up and radiation damage will introduce defects into the material that could have a significant impact on tritium release. Therefore, due to the importance of point defects for controlling tritium mobility through the bulk crystal, this work focuses on understanding the intrinsic defect chemistry of Li_8_PbO_6_ using a point defect model similar to the works of Murphy and Hine.^10,11^ Although due to the high temperatures expected during reactor operation, thermodynamic contributions may become a significant contributor toward the defect composition. Thus, we also present in this work a modified method to include temperature incorporation into the defect chemistry by calculating the constituent chemical potentials used in the defect formation energy calculations from the *T*-dependent Gibbs free energies of the reactants.

## Crystallography

**Figure 1:**
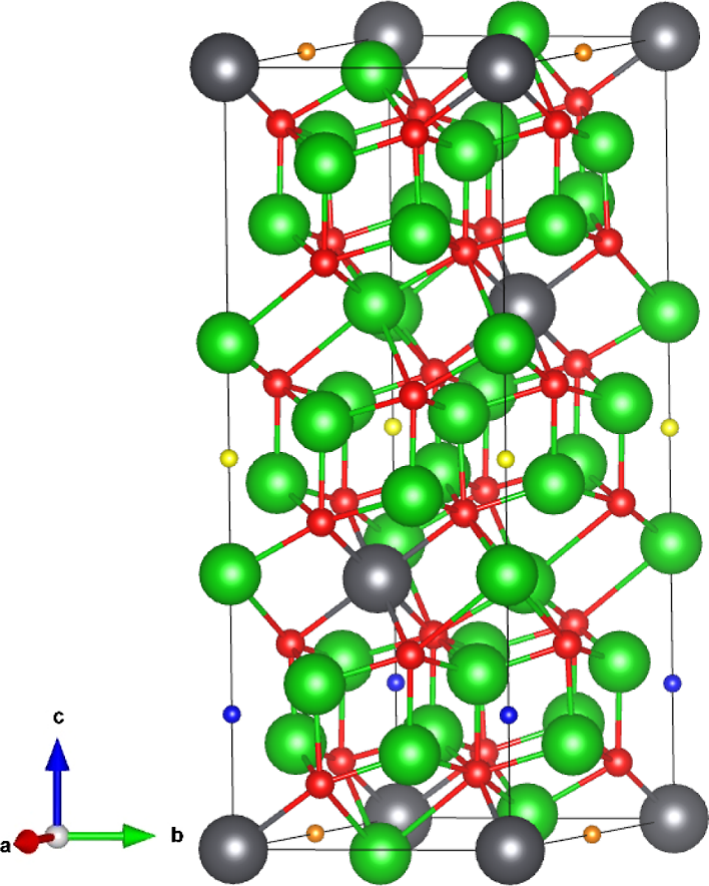
Structure of 45-atom unit cell of Li_8_PbO_6_. Green spheres represent Li ions, red spheres represent O ions, and the gray spheres represent Pb ions. Interstitial sites 1–3 are illustrated as orange, blue, and yellow and ions, respectively. Not all possible interstitial sites are shown in the figure.

Li_8_PbO_6_ adopts the trigonal *R*3̅*H* [148] space group.^12^ Within the unit cell, Li occupies two symmetrically distinct Wyckoff positions, which are the tetragonally coordinated 18f sites and the octahedrally coordinated 6c sites. The O and Pb ions occupy the 18f and 3a Wyckoff positions, respectively. Li_8_PbO_6_ adopts a complex layered structure alternating between a pure Li layer and a mixed Li–Pb layer in the following sequence: PbLi_2_–O_3_–Li_3_–Li_3_–O_3_. In the pure Li layers, Li occupies the tetrahedrally coordinated site exclusively, whereas in the mixed Li–Pb layer, Li occupies the octahedrally coordinated site. For the remainder of this paper, the tetragonally coordinated Li will be referred to as Li_1_ and the octahedrally coordinated Li will be referred to as Li_2_. Three symmetrically distinct interstitial sites were identified in Li_8_PbO_6_. The coordinates of the interstitial sites are presented in Table 1 and the sites are indicated in Figure 1.

**Table 1:** Interstitial Sites in Li_8_PbO_6_ are in Fractional Coordinates

interstitial site	*x*	*y*	*z*
1	0.25	0	0
2	0	0	0.17275
3	0	0	0.5

## Methodology

### Defect Formalism

Key to understanding how tritium retention may change during operation is understanding how the defect population will evolve. As discussed above, the defect chemistry of ceramic breeder materials will be modified due to lithium burn-up and radiation damage. The population of defects due to radiation damage cannot be quantified using thermodynamics and will be addressed in future work; therefore, we focus on understanding the underlying defect population due to the incorporation of nonstochiometry arising from the burn-up of the lithium.

The charge neutrality condition requires an overall balance between the ionic and electronic defects in the system, i.e.
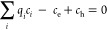
4where *c*_*i*_ is the concentration of any point defect, *i*, with a charge *q*_*i*_, and *c*_e_ and *c*_h_ are the concentrations of electrons in the conduction band and holes in the valence band, respectively.

#### Point Defect Concentrations

The concentration of any point defect in a material is related to the Gibbs formation energy according to
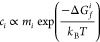
5where *m*_*i*_ is the multiplicity of the defect (i.e., the number of possible sites for defect, *i*, per formula unit), *k*_B_ is the Boltzmann constant, and *T* is the temperature.

It is typically assumed that the vibrational contributions to the free energy of a solid are negligible and so the Gibbs formation energy can be approximated to be the change in internal energy change associated with the formation of the defect *i* using the formalism of Zhang and Northup^13^
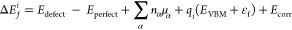
6where *E*_defect_ and *E*_perfect_ are the DFT total energies of the supercell with and without the presence of the point defect, respectively, *n*_α_ is the number of atoms, of species α, added/removed from the supercell to construct the defect, μ_α_ is the chemical potential of the species α, *q*_*i*_ is the charge of the defect *i*, *E*_VBM_ is the valence band maximum (VBM) of the defect-free system, ε_f_ is the Fermi energy, and *E*_corr_ is a charge correction term used to mitigate for finite-size effects.

To calculate the chemical potentials of the constituents of Li_8_PbO_6_, we first note Li_8_PbO_6_ can be formed from the two binary oxides Li_2_O and PbO_2_ (as is done by Colominas et al.^14^) via the reaction
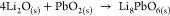
7

For any condition, the sum of the chemical potentials of the constituents must equal the chemical potential of Li_8_PbO_6_
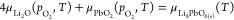
8where μ_Li_2_O_(*p*_O_2__,*T*) and μ_PbO_2__(*p*_O_2__,*T*) are the chemical potentials of Li_2_O and PbO_2_ as a function of oxygen partial pressure and temperature, respectively, and  is the chemical potential of solid Li_8_PbO_6_.

At low temperatures, vibrational contributions to the constituent chemical potentials can be safely assumed to be negligible, and so . However, for breeder blankets that typically operate at high temperatures, vibrational and entropic factors can be expected to be present. Therefore, in this paper, we examine the impact of including these contributions into the energies of the reference states for the solid species by including the temperature dependence of the DFT terms in eqs 9 and 10, i.e., , μ_PbO_2_(s)_^DFT^(*T*), and μ_Li_8_PbO_6_(s)_^DFT^(*T*).

The chemical potentials of the constituent oxides are bound by the Gibbs energy of competing phases. If the chemical potential exceeds this upper bound, it becomes thermodynamically favorable for the precipitate to form. At the Li_2_O saturation limit, or Li_2_O-rich conditions, the chemical potential of the binary oxide can be determined using DFT as
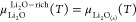
9

To calculate the lower bound for the chemical potential of each binary compound, we assume that the other component must be at their upper bound
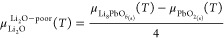
10

The values for the chemical potentials can fall anywhere in this rich/poor range; therefore, we define a fraction, *f*, for each oxide which controls, where in this range, the chemical potential falls
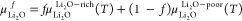
11

The fraction, *f*, assigned to each oxide is constrained by the following equation
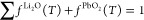
12

The two constituent oxides can also be decomposed to their subcomponents to determine the chemical potentials of the elements Li, Pb, and O. Using Li_2_O once again as an example
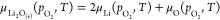
13where μ_Li_(*p*_O_2__,*T*) and μ_O_(*p*_O_2__,*T*) are the chemical potentials of Li and O in Li_2_O. Rather than determining the chemical potential of oxygen from the O_2_ molecule directly from DFT, which is problematic due to self-interaction, we adopt the method of Finnis et al.^15^ and use the experimental formation energy of the oxide compound
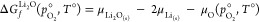
14where  is the known experimental formation energy for Li_2_O, taken as −6.205, and −2.845 eV for PbO_2_ according to Chase.^16^ μ_Li(s)_ is the chemical potential of Li metal, obtained with DFT. This method results in  being calculated twice, once for each oxide. A final value of  is calculated as a weighted average of the contributions by the fractions of oxides present
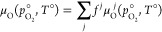
15

The temperature and pressure dependence of the oxygen chemical potential cannot be neglected and is extrapolated from  using the relationship
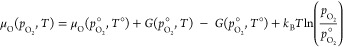
16where  is determined from the real heat capacities for the O_2_ molecule, taken from the NIST Chemistry WebBook.^17^

#### Electronic Defects

The concentrations of electronic defects in the system can be determined from Fermi–Dirac statistics according to
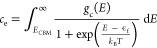
17and
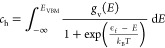
18where *g*_c_(*E*) and *g*_v_(*E*) are calculated from the electronic density of states for Li_8_PbO_6_ using the hybrid functional of Heyd, Scuseria, and Ernzerhof (HSE06),^18^ and *E*_VBM_ and *E*_CBM_ are the energies of the valence band maximum and the conduction band minimum, respectively. Details on the density of states calculation can be found in our previous work on the fundamental properties of Li_8_PbO_6_.^19^

### Computational Procedure

DFT simulations were performed using the Vienna ab initio Simulation Package (version 5.4.4) plane-wave pseudopotential code,^20^ employing projector augmented wave pseudopotentials.^21^ All defect simulations utilized the generalized gradient approximation of Perdew, Burke, and Ernzerhof.^22^ Integration over the Brillouin zone was performed using a Monkhorst–Pack grid^23^ with a separation between *k*-points of 0.0316 × 2π Å^–1^ along the *x* and *y* axes and 0.0344 × 2π Å^–1^ along the *z*-axis. The plane-wave cutoff energy was set to 650 eV, the energy threshold for electronic convergence is set as 10^–8^ eV, and structural convergence was deemed complete when the forces on all atoms did not exceed 0.01 eV Å ^–1^. Defects are inserted into a supercell constructed from 2 × 2 × 1 repetitions of the 45-atom, 5.58 × 5.58 × 15.81 Å^–1^ unit cell, resulting in a supercell containing 180 atoms. Defect charges are modeled by adding or removing electrons from the supercell. It is noted that the efficacy of the computational model is established in previous work.^19^

#### Finite Size Effects

The introduction of charged defects into relatively small supercells in DFT results in the presence of finite size artifacts. The main source of these artifacts is the Coulombic interaction between the defect and its periodic image and the neutralizing background charge. Finite size effects are accounted for in the defect formation energy via implementation of a charge correction term, labeled *E*_corr_ in eq 6.

In this paper, the anisotropic screening correction developed by Kumagai and Oba^24^ is utilized, which builds on the point charge correction developed by Freysoldt, Neugebauer and Van de Walle (FNV).^25^ Kumagai and Oba build on the method by utilizing atomic site potentials instead of planar averaged electrostatic potentials utilized by FNV. Using the atomic site electronic potentials of supercells with (*V*_def,q_) and without (*V*_perf_) defects, *E*_corr_ for a defect with charge *q* is calculated
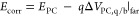
19
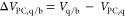
20
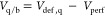
21Δ*V*_PC,q/b_ is the potential difference between the defect induced potential (*V*_q/b_) and the point charge (PC) potential, *V*_PC,q_. Δ*V*_PC,q/b_|_far_ is Δ*V*_PC,q/b_ at a position far from the defect site but still within the supercell. *E*_PC_ is the PC correction, calculated for each charged defect using eq 22
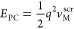
22where *v*_M_^scr^ is the Madelung potential for a point charge in a general three-dimensional box screened by a general dielectric. Taking into account the anisotropic dielectric properties of Li_8_PbO_6_, we calculate a value of *v*_M_^scr^ using the system’s dielectric tensor, ϵ̅, and the method described by Murphy and Hine^26^
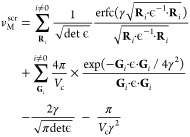
23where the sum extends over all vectors of the direct (**R**_*i*_) and reciprocal (**G**_*i*_) lattices, γ is a suitably chosen convergence parameter, and *V*_c_ is the volume of the supercell. The dielectric tensor is taken from our previous study examining the fundamental properties of Li_8_PbO_6_.^19^
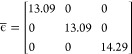
24

#### Phonon Calculations

The Gibbs free energies of Li_2_O, PbO_2_, and Li_8_PbO_6_ can be obtained from the dynamical matrix. Gibbs free energies for Li_8_PbO_6_ and the reactant PbO_2_ are taken from our previous works examining thermodynamic properties of octalithium ceramics^27^ using the density functional perturbation theory (DFPT) code phonopy^28^ that employs the QHA.^29^ QHA calculations were performed for additional binary and ternary compounds in the Li–Pb–O phase group [Li_2_PbO_3_, PbO-M (orthorhombic), PbO-L (tetragonal), and Pb_3_O_4_], with a *k*-point separation of 0.2–0.3 × 2π Å^–1^, and using 11 different volumes in equal steps of 3% within a range of ±15% of the respective relaxed unit cell. The energy for Li_2_O is taken from specific heat capacities obtained using a combination of Chase^30^ and Johnston and Bauer^31^ due to the presence of phonon–phonon interactions in Li_2_O which cause an overestimate in DFPT-calculated heat capacities in FCC lattice structures.^32^ Due to computational limitations with performing DFPT simulations of point defects (particularly due to the asymmetry introduced), DFPT simulations for the point defects were not performed in this paper.

#### Defect Analysis

We investigate vacancy-, interstitial-, and antisite-type intrinsic defects in Li_8_PbO_6_. All charge states that are reasonably attainable are studied for each of these defects and a full list of the defects considered in this study are presented in Table 2, using Kröger–Vink notation, modified to display relative charge as an integer value.^33^

**Table 2:** Defect Formation Energies at the Valence Band Maximum at 1000 K under Li_2_O-Rich Conditions with an Oxygen Partial Pressure of 0.2 atm^a^

	defect	*E*_f_ (eV)	*E*_f_ (*T*) (eV)		defect	*E*_f_ (eV)	*E*_f_ (*T*) (eV)
1	*V*_Li1_^–1^	2.261	1.909	29	Li_*i*3_^0^	2.509	2.861
2	*V*_Li1_^0^	1.179	0.827	30	Li_*i*3_^1^	–0.826	–0.474
3	*V*_Li2_^–1^	2.663	2.312	31	Li_split_^0^	2.849	3.201
4	*V*_Li2_^0^	1.526	1.175	32	Li_split_^1^	–0.433	–0.081
5	*V*_Pb_^–4^	10.238	8.698	33	Pb_*i*1_^0^	8.773	10.314
6	*V*_Pb_^–3^	8.110	6.569	34	Pb_*i*1_^1^	5.988	7.529
7	*V*_Pb_^–2^	6.514	4.974	35	Pb_*i*1_^2^	3.142	4.682
8	*V*_Pb_^–1^	5.376	3.835	36	Pb_*i*1_^3^	1.822	3.363
9	*V*_Pb_^0^	4.968	3.427	37	Pb_*i*1_^4^	0.259	1.800
10	*V*_O_^0^	2.870	2.870	38	Pb_*i*2_^0^	6.799	8.340
11	*V*_O_^1^	0.484	0.484	39	Pb_*i*2_^1^	3.950	5.491
12	*V*_O_^2^	–1.813	–1.813	40	Pb_*i*2_^2^	1.108	2.649
13	Pb_Li1_^0^	4.939	6.128	41	Pb_*i*2_^3^	–0.316	1.225
14	Pb_Li1_^1^	1.638	2.827	42	Pb_*i*2_^4^	–1.766	–0.225
15	Pb_Li1_^2^	–0.102	1.087	43	Pb_*i*3_^0^	8.021	9.562
16	Pb_Li1_^3^	–1.789	–0.600	44	Pb_*i*3_^1^	4.696	6.236
17	Pb_Li2_^0^	3.964	5.154	45	Pb_*i*3_^2^	1.544	3.084
18	Pb_Li2_^1^	0.689	1.879	46	Pb_*i*3_^3^	–0.025	1.515
19	Pb_Li2_^2^	–1.162	0.027	47	Pb_*i*3_^4^	–1.640	–0.099
20	Pb_Li2_^3^	–2.980	–1.791	48	O_*i*1_^–2^	8.181	8.181
21	Li_Pb_^–3^	7.435	6.246	49	O_*i*1_^–1^	4.751	4.751
22	Li_Pb_^–2^	5.651	4.461	50	O_*i*1_^0^	1.739	1.739
23	Li_Pb_^–1^	4.363	3.175	51	O_*i*2_^–2^	7.601	7.601
24	Li_Pb_^0^	3.640	2.451	52	O_*i*2_^–1^	5.057	5.057
25	Li_*i*1_^0^	3.006	3.358	53	O_*i*2_^0^	3.578	3.578
26	Li_*i*1_^1^	–0.305	0.046	54	O_*i*3_^–2^	7.789	7.789
27	Li_*i*2_^0^	2.666	3.018	55	O_*i*3_^–1^	4.985	4.985
28	Li_*i*2_^1^	–0.605	–0.253	56	O_*i*3_^0^	3.553	3.553

a The *E*_f_ values are those where the energy minimized values for the reference states are employed and *E*_f_ (*T*) have temperature effects incorporated into the reference states for the solids. Note that for the oxygen defects, the values are identical as the chemical potentials for oxygen already included a temperature contribution.

Final calculations of the defect concentrations at a given temperature and oxygen partial pressure can be obtained by determining the Fermi energy that achieves charge neutrality according to eq 4. The Fermi level is determined using a linear bisection approach in the Defect Analysis Package (DefAP).^34^

## Results and Discussion

### Defect Formation Energies

In the following subsection, we examine defect formation energies under Li_2_O-rich conditions with an oxygen partial pressure of 0.2 atm at 1000 K. Defect formation energies for all possible intrinsic point defects and their respective charge states at the valence band maximum have been included in Table 2.

**Figure 2:**
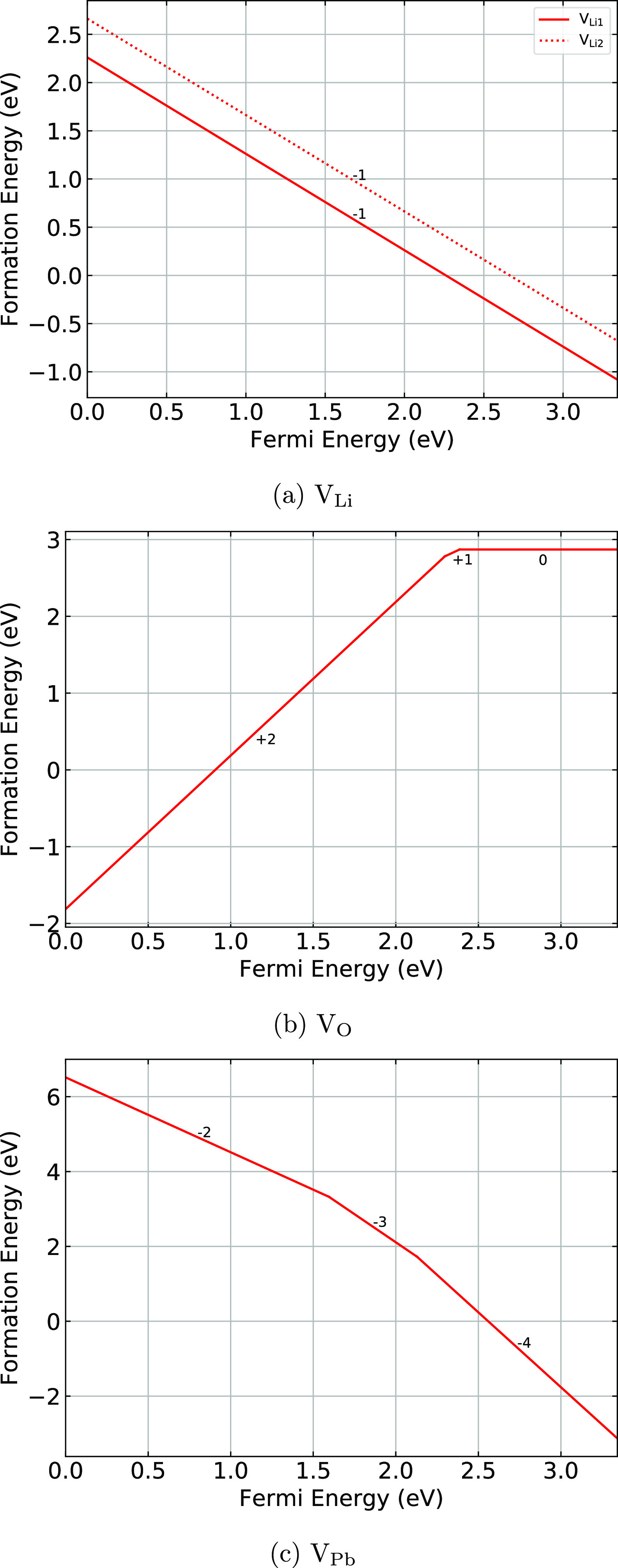
Formation energies of vacancy defects as a function of the Fermi energy.

Figure 2 shows the formation energies of the vacancy defects in Li_8_PbO_6_ as a function of the Fermi energy. Lithium vacancy defects for both unique lattice sites occupy the −1 charge state across the entire band gap with the Li1 site having a lower formation energy, as seen in Figure 2a. This suggests that the majority of lithium vacancy defects will occur at the tetrahedrally coordinated 18f Wyckoff position, rather than the octahedral 6c site. This is simply due to the requirement to break fewer bonds to remove the tetrahedrally coordinated atom.

The formation energies for the oxygen and lead vacancy defects as a function of the Fermi level are presented in Figure 2b,c. At the valence band maximum, the oxygen vacancy is predicted to be in the +2 charge state. The same charge state is predicted to dominate for the majority of the band gap. At roughly 2.3 eV there is a transition to the +1 charge state, although the region where this state dominates is relatively small. As the Fermi level approaches the conduction band, the charge neutral oxygen vacancy dominates. For the lead vacancy defect (Figure 2c) only the highly charged states −2, −3, and −4 are predicted to be stable across the width of the band gap.

**Figure 3:**
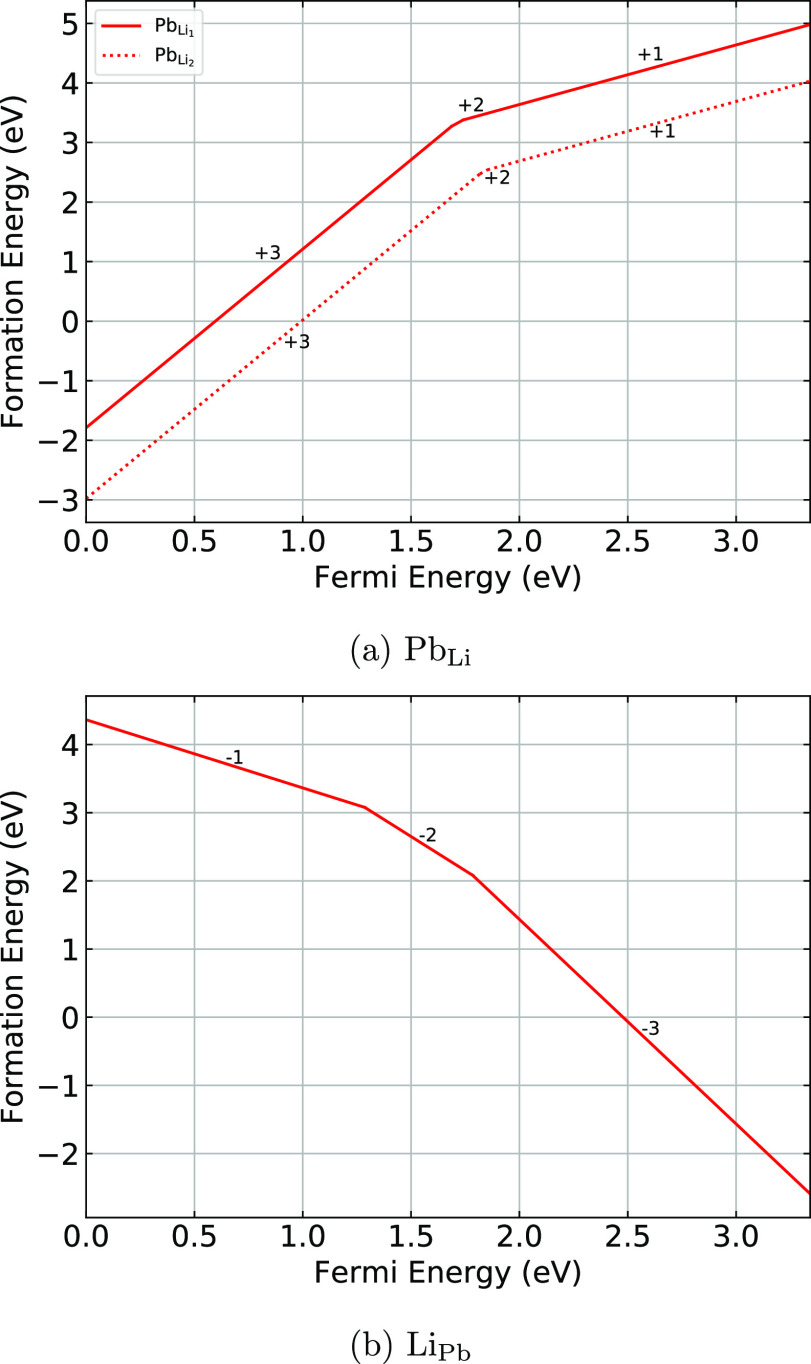
Formation energies of antisite defects as a function of the Fermi energy.

Figure 3 shows the formation energies for the antisite defects in Li_8_PbO_6_ (Pb_Li_ and Li_Pb_, respectively). Lead may substitute for lithium on either of the two symmetrically distinct sites. Both sites show broadly the same distribution of charge states as a function of Fermi Energy. Figure 3a suggests that it is thermodynamically preferable for lead to occupy the octahedrally coordinated 6c position in the mixed Li–Pb layer, rather than the pure lithium layer, due to the greater number of nearest neighbor oxygen ions. Pb_Li_ does not appear to occupy the charge-neutral state and instead broadly favors the +3 and +1 states at the valence band maximum and conduction band minimum, respectively, with some minor transitional occupation of the +2 state. The dominance of the +3 and +1 defect charge states corresponds to lead in the +4 and +2 oxidation states occupying a V_Li_^1–^ vacancy. For lithium substitution onto the lead site, the −1 charge state is predicted to dominate at the valence band maximum, while the −3 charge state dominates at the conduction band minimum. The transitions between charge states occur in very similar positions in the band gap to the lead vacancy defect.

**Figure 4:**
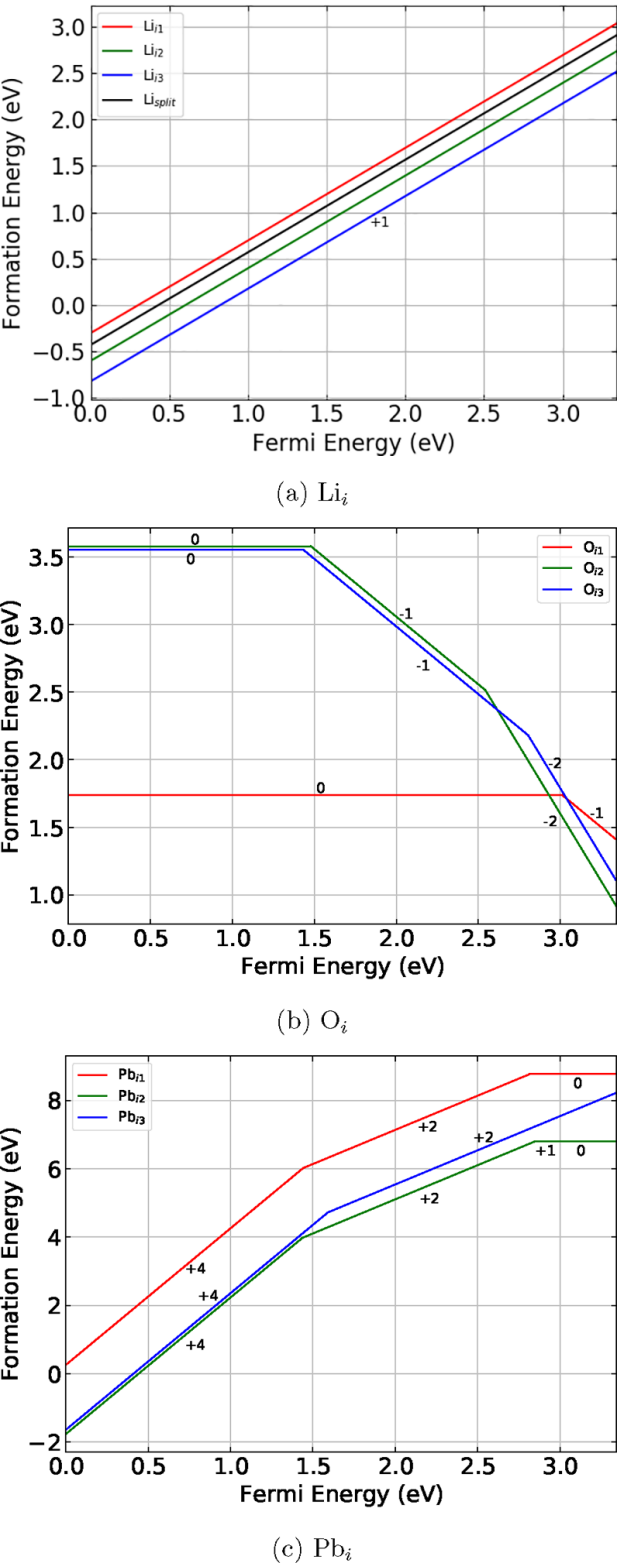
Formation energies for interstitial defects as a function of the Fermi energy.

**Figure 5:**
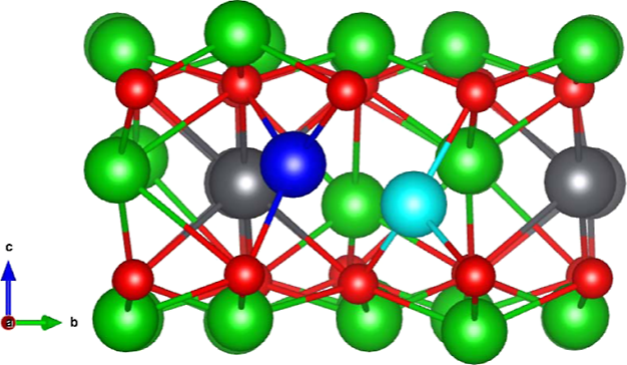
Li split interstitial defect in the mixed Li–Pb plane. The blue sphere represents the lithium ion placed into the unit cell, which forms a split interstitial with the displaced lithium ion represented using the teal sphere.

Figure 4 shows the formation energies for the interstitial defects as a function of the Fermi energy. Lithium interstitial defects (Figure 4a) are predicted to exist in the +1 charge state across the entire width of the band gap. Li interstitial defects are predicted to be most favorable on sites 2 and 3, which are located between the two pure Li planes. By contrast, interstitial site 1, which lies in the mixed Li–Pb plane, is shown to have a high formation energy, which is likely due to the proximity to a positively charged lead ion. It should be noted that a split interstitial site was found in the Li interstitial case within the mixed Li–Pb layer, distinct from site 1. The site is visualized in Figure 5.

As shown in Figure 4b, the oxygen interstitial defects exhibit significantly different behaviors depending on the site they occupy. The most favorable interstitial site for oxygen to occupy was found to be site 1 in the mixed Li–Pb plane, where the charge-neutral defect dominates across nearly the entire width of the band gap, with some occupation of the −1 state toward the edge of the conduction band. Sites 2 and 3 exhibit a similar trend in formation energies as a function of the Fermi Energy, with the −1 and charge-neutral states dominating across the majority of the band gap and the −2 states only becoming important close to the conduction band.

For the lead interstitial defect, it is predicted that site 2 is the most thermodynamically stable. For all three sites, the majority of the band gap is occupied by the +4 and +2 charge states, indicating depopulation of the outermost s orbitals in both cases, and both p and s orbitals in the +4 state. For site 3, the +2 charge state extends up to the conduction band minimum, whereas for sites 1 and 2, there are transitions to lower charge states evident.

Almost all defects shown in Figure 4 occupy primarily their formal charge states, with the exception of an oxygen interstitial defect that appears to have a slight preference for the −1 state. As most defects occupy their formal charge states, there will be few states in band gap and, consequently, the widely known self-interaction error will not significantly impact the defect chemistry predicted here,^35^ especially as a charge-correction is used within the model to account for this potentiality.

### Temperature of Stable Phases

Due to the lack of literature available on the stability of Li_8_PbO_6_, phase diagrams were constructed to explore stable regions in the Li–Pb–O system. Compounds examined other than Li_8_PbO_6_ include Li_2_O and PbO_2_ (both reactants used in the sintering process for Li_8_PbO_6_), Li_2_PbO_3_, and Li_4_PbO_4_ (a trace compound found during sintering). Alternative binary oxide phases in the Li–O^36^ (Li_2_O_2_ and LiO_2_) and Pb–O^37^ (PbO-M, PbO-L, and Pb_3_O_4_) groups have been considered, although the alternative binary oxide phases are not shown to have a significant impact on the phase stability region of Li_8_PbO_6_, particularly alternative Li–O phases, as under the conditions considered for this work, Li_2_O is predicted to be the most thermodynamically preferred phase.^38^

**Figure 6:**
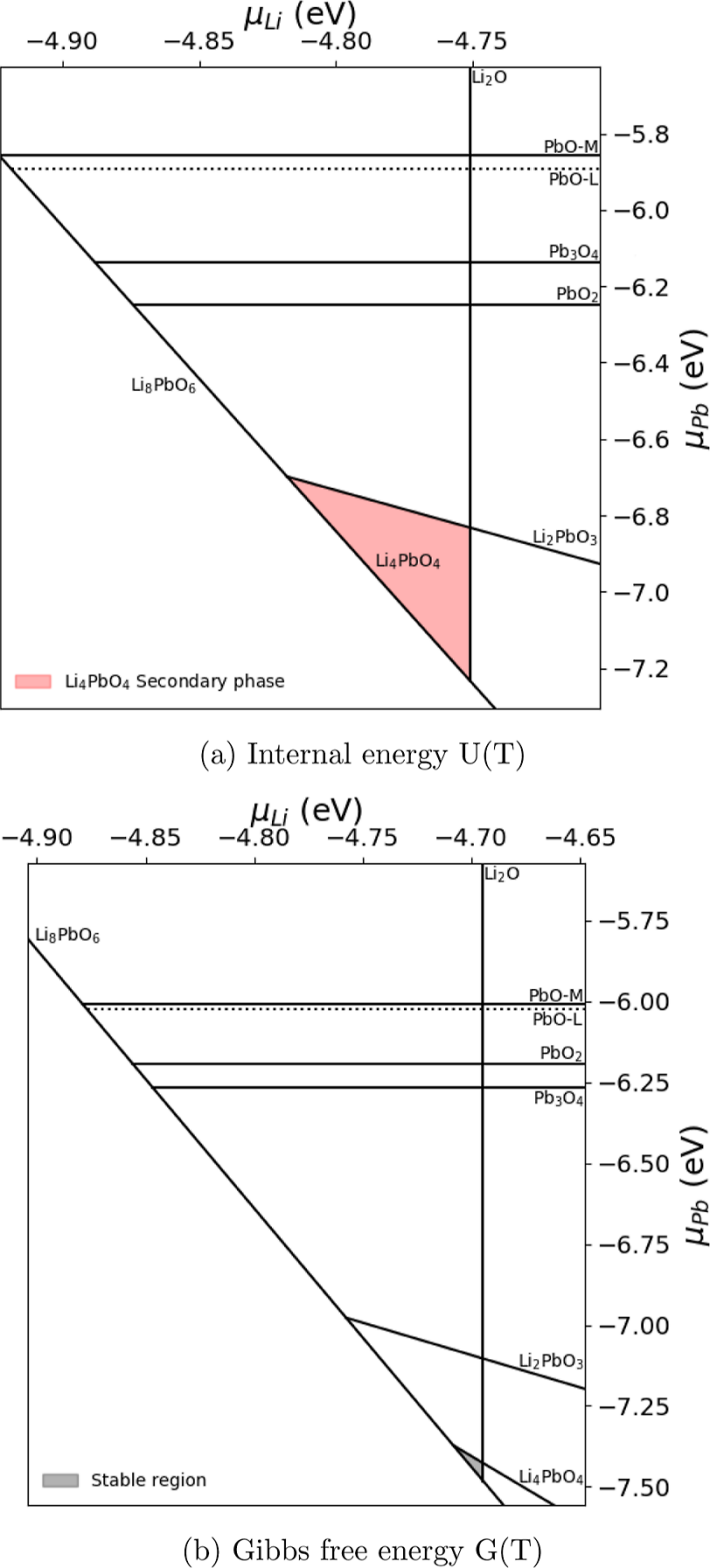
Phases of the Li–Pb–O system as a function of Li and Pb chemical potentials using internal (a) and Gibbs free energies (b), respectively. Each line represents the minimum boundary of ∑_*i*_μ_*i*_ where the respective compound is stable. The gray shaded region in (b) illustrates the phase space where the formation is exclusively Li_8_PbO_6_ over any other phase [i.e., *E*(Li_8_PbO_6_) < ∑_*i*_μ_*i*_, ∀ *E*(compound ≠ Li_8_PbO_6_)], and the red region in (a) shows the region where Li_4_PbO_4_ is a secondary phase to Li_8_PbO_6_. The dotted line represents PbO-L to better distinguish between PbO phases. *T* = 300 K, oxygen partial pressure (OPP) = 0.2 atm,  = −4.78 eV.

**Figure 7:**
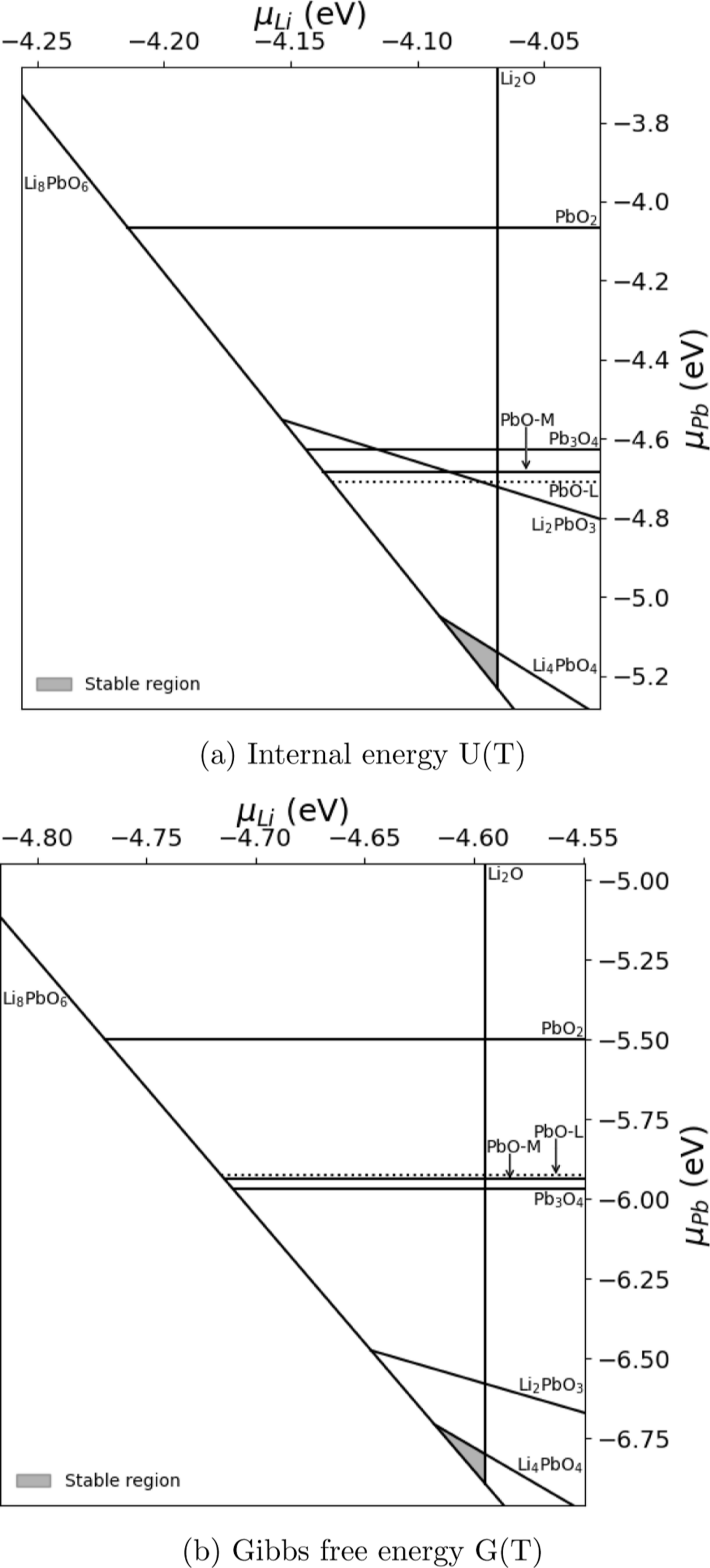
Stable phases of the Li–Pb–O system as a function of Li and Pb chemical potentials using internal (a) and Gibbs free energies (b), respectively. *T* = 1000 K, OPP = 0.2 atm,  = −5.60 eV.

In this paper, a comparison is made between the use of the internal and Gibbs free energies for the binary and ternary compounds in the Li–Pb–O system in the determination of phase stability. The alternative Pb–O phases have been included in the phase stability diagrams presented in Figures 6 and 7.

For low temperatures, there is a clear distinction between plots given in Figure 6 as to whether there exists a region in which Li_8_PbO_6_ is stable. If the Gibbs free energy is not taken into account in the constituent compounds Li_2_O and PbO_2_, Li_8_PbO_6_ is deemed unstable, with significant quantities of Li_4_PbO_4_ and Li_2_PbO_3_ appearing within the system. By contrast, using the Gibbs free energies there is a small stable region for Li_8_PbO_6_ where no formation of secondary phases is expected, suggesting if accounted for, Li_8_PbO_6_ may form at 300 K. The stable region is bordered by boundaries of Li_4_PbO_4_ and Li_2_O regions so the presence of trace quantities of Li_2_O and Li_4_PbO_4_ is likely. Aside from a single paper exploring the sintering process for Li_8_PbO_6_ from Li_2_O and PbO_2_ by Colominas et al.,^14^ there is a lack of literature on fabrication of Li_8_PbO_6_, with which to compare our results.

For high temperatures (Figure 7), there is a stark difference between the internal and Gibbs free energy phase diagrams. At 1000 K, both the massicot and litharge phases of PbO and Pb_3_O_4_ are predicted to have more favorable internal energies compared to PbO_2_, as expected. Although Pb_3_O_4_ is predicted to have a slightly lower Gibbs formation energy compared to PbO. The stability region for Li_8_PbO_6_ is not impacted by any of the alternative phases excluding Li_4_PbO_4_, assuring the secondary formation of trace Li_4_PbO_4_ as seen in Colominas et al.^14^ Unfortunately, the exclusive region for Li_8_PbO_6_ formation in the chemical potential phase space is relatively quite small, regardless of the choice of method, and it is expected the formation of Li_8_PbO_6_ will thus only occur close to the Li_2_O-rich limit.

### Intrinsic Defect Chemistry

Having defined the region of the chemical potential space where Li_8_PbO_6_ is thermodynamically stable, we now explore the defect chemistry of the material in this region. Figures 6 and 7 show that the Li_8_PbO_6_ phase is not stable at the PbO_2_ saturation limit, due to the formation of Li_4_PbO_4_ and Li_2_PbO_3_ phases. By contrast, there is a region where the octalithium compound is stable at the Li_2_O saturation limit. Therefore, this region is explored first. A comparison is made between the predicted defect chemistry where the temperature effects are included in the energies for the references states and where they are not. This is important due to the high operational temperatures anticipated in breeder blanket materials, which have a maximum operational temperature of 920 °C for the ceramic in HCPB designs.^39^

**Figure 8:**
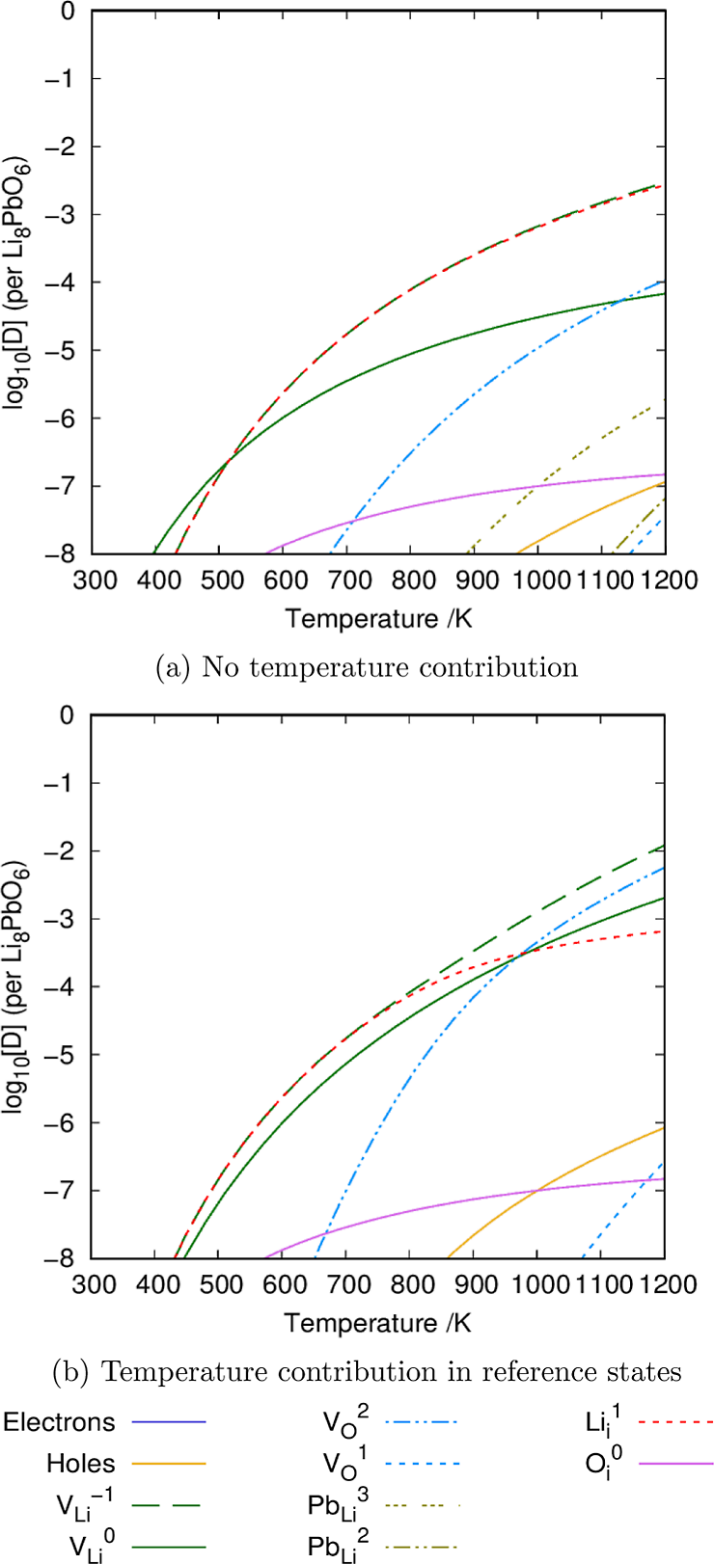
Defect chemistry of Li_8_PbO_6_ under Li_2_O-rich conditions ignoring and incorporating temperature contributions to the energies of the reference states, respectively. OPP = 0.2 atm.

Under Li_2_O-rich conditions (Figure 8), charged lithium vacancy defects are the dominant type of defect across the entire temperature range, which is significant as the lithium vacancy may act as a trapping site for tritium.^40^ The method of charge compensation, however, is shown to be different when including temperature effects for the reference states. If temperature effects are neglected, charge compensation is provided by oppositely charged lithium interstitials for the entire temperature range. The Li_*i*_^+1^ is also predicted to provide charge balance at low temperatures (<980 K) when the reference states are modified to include temperature. By contrast, for high temperatures (>980 K), incorporating temperature into the energies for reference states changes the charge compensation mechanism to the V_O_^+2^ defect. Furthermore, the introduction of temperature effects also reduces the overall concentration of lead-containing defects at a high temperature. Overall, the modification of the defect chemistry due to incorporating temperature into the reference states demonstrates the importance of including these for the study of materials operating at high temperatures.

### Oxygen Partial Pressure

**Figure 9:**
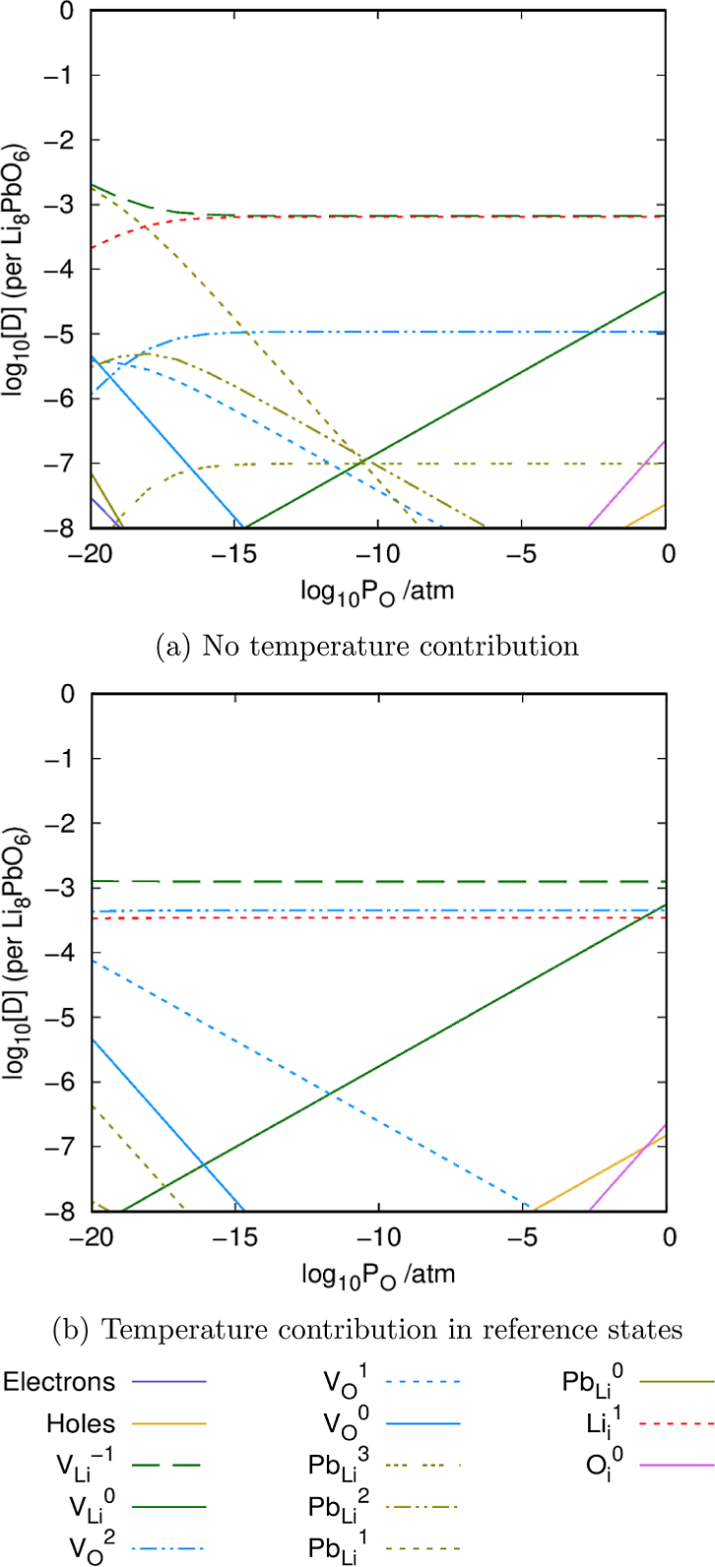
Oxygen partial pressure dependence of intrinsic defects in Li_8_PbO_6_ under Li-rich conditions ignoring and incorporating temperature contributions to the energies of the reference states, respectively. The temperature is fixed to 1000 K.

Next, we examine the dependence of the defect chemistry on the oxygen partial pressure at the Li_2_O saturation limit in Li_8_PbO_6_ at high temperatures using both procedures in Figure 9.

Figure 9 further highlights the differences that arise due to the incorporation of temperature effects in a more complete manner. When temperature contributions to the reference states are ignored the lithium vacancy defect is expected to be dominant across the partial pressure range. At low oxygen partial pressures, charge compensation is provided by the Pb_Li_^1^ defect. At higher partial pressures, there is a transition to a point where the Li_*i*_^1^ defect provides charge compensation. This is a marked contrast to what is predicted if the temperature contributions are added. This model predicts that the V_Li_^–1^ defect is dominant, with charge compensation coming from a combination of the V_O_^+2^ and Li_*i*_^+1^ defects.

### Dominant Defects in Stable Regions

**Figure 10:**
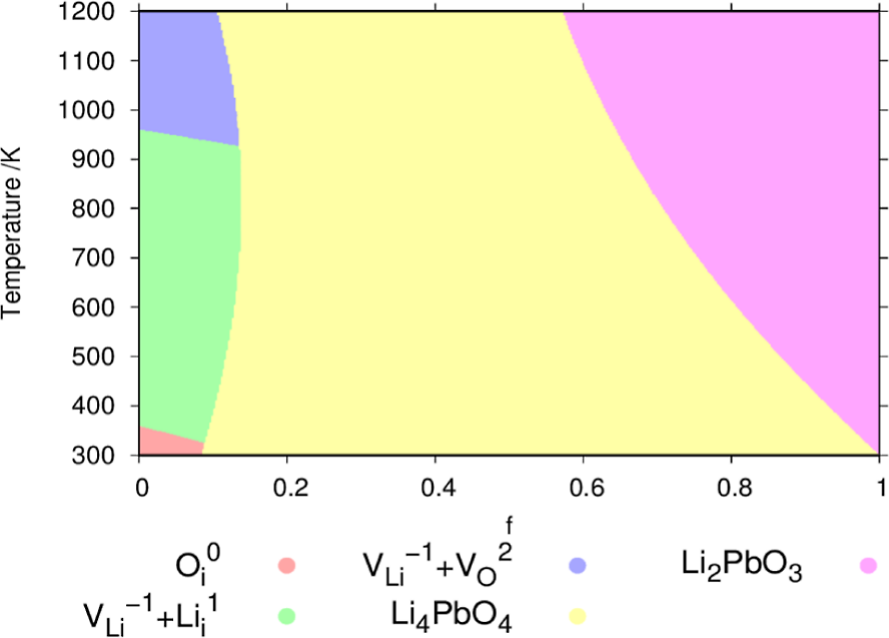
Phase diagram illustrating stable regions for the Li–Pb–O ternary system as a function of stoichiometry and temperature. 0 represents the Li_2_O-rich limit and 1 represents that for PbO_2_. The yellow and pink regions represent areas where a competing Li–Pb–O ternary compound becomes the dominating phase. Regions occupied by Li_8_PbO_6_ are shown as the dominant defect predicted in the respective region. OPP = 0.2 atm.

In this section, we explore the defect chemistry of Li_8_PbO_6_ away from the saturation limit for Li_2_O by plotting the dominant defects or compounds as a function of the rich/poor fraction, *f* (see eq 11) and the temperature in Figure 10.

Throughout this subsection, only defect chemistry was predicted when temperature effects for the binary and ternary compounds are presented.

As expected from Figures 6 and 7, the only region where Li_8_PbO_6_ is predicted to dominate over competing phases is close to the Li_2_O saturation limit. Under a 4:1 ratio of Li_2_O/PbO_2_, Li_4_PbO_4_ is expected to be the most stable phase, and at the PbO_2_ limit, the Li_2_PbO_3_ phase is the most stable, particularly for higher temperatures. The narrow width of the dominant region for Li_8_PbO_6_ may place a limit on the operational lifetime of the material when employed as a ceramic breeding material as the quantity of Li that can undergo transmutation before the material undergoes a phase change is quite low.

Inspecting the dominant defects in the Li_8_PbO_6_ phase, it is clear that the charged lithium vacancy defect dominates almost the entire defect profile with a small region where oxygen interstitial defects become the most common defect at room temperature. The primary charge-compensation mechanism is the Li_*i*_^1^ lithium interstitial, followed by the V_O_^2^ vacancy at high temperatures. Due to the Li_8_PbO_6_ phase only being stable close to the Li_2_O saturation limit, the defect chemistry of Li_8_PbO_6_ is essentially very similar to that of the Li_2_O-rich material (Figures 8 and 9).

**Figure 11:**
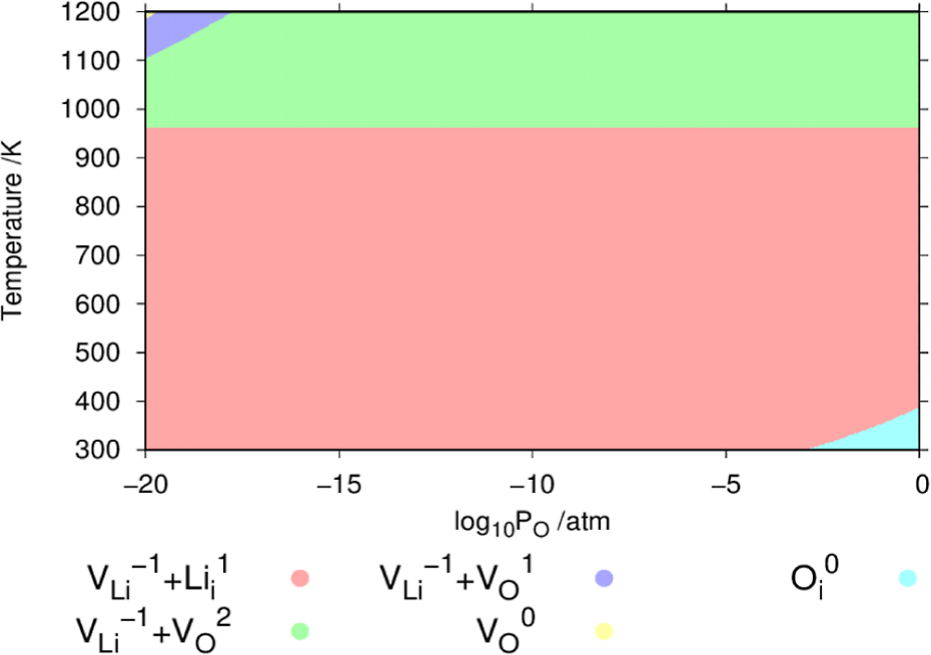
Phase diagram illustrating regions for predictions of the dominant defect in the temperature-oxygen partial pressure space under Li_2_O-rich conditions.

As illustrated in Figure 11, regardless of the choice of oxygen partial pressure in the atmosphere and temperature, the V_Li_^–1^ defect is predicted to be the dominant defect for nearly the entire phase space, with the primary charge-compensation mechanism being the Li_*i*_^1^ interstitial at low-moderate temperatures and V_O_^2^ at high temperatures above roughly 960 K, where the change in charge-compensation mechanism for the V_Li_^–1^ defect to V_O_^2^ acts almost completely independently of oxygen partial pressure.

At low oxygen partials and high temperatures, the concentration of V_O_ defects increases. Initially charge-compensating for the lithium vacancies in the form of V_O_^2^ defect, followed by a transition where the V_O_^1^ defect becomes the most common as temperature increases at low oxygen partial pressure before finally, in the extreme case, a small region appears at concentrations of 10^–20^ atm and at 1200 K where V_O_^0^ begins to dominate.

### Li Burn-Up

**Figure 12:**
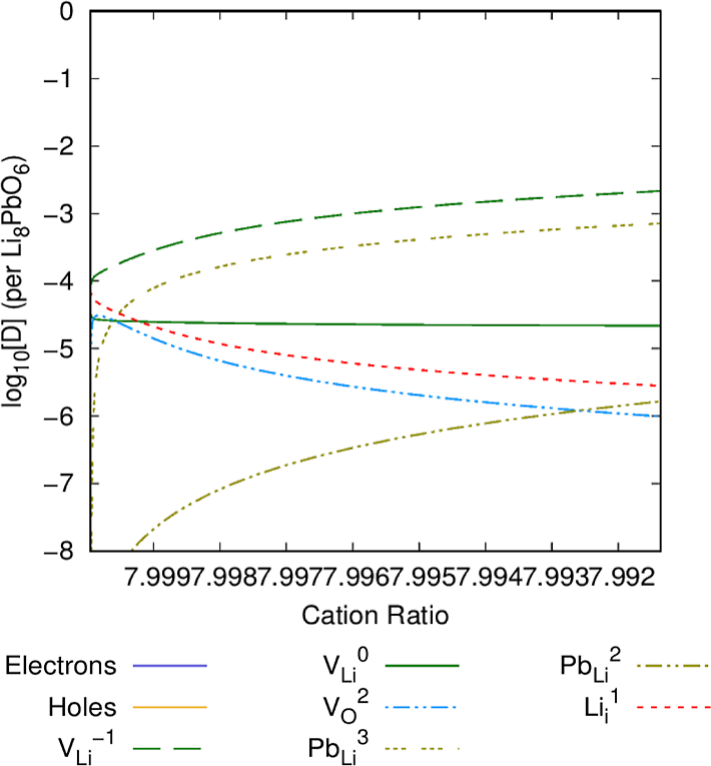
Intrinsic defect concentration in Li_8_PbO_6_ as a function of lithium burn-up. *T* = 800 K and OPP = 0.2 atm.

**Figure 13:**
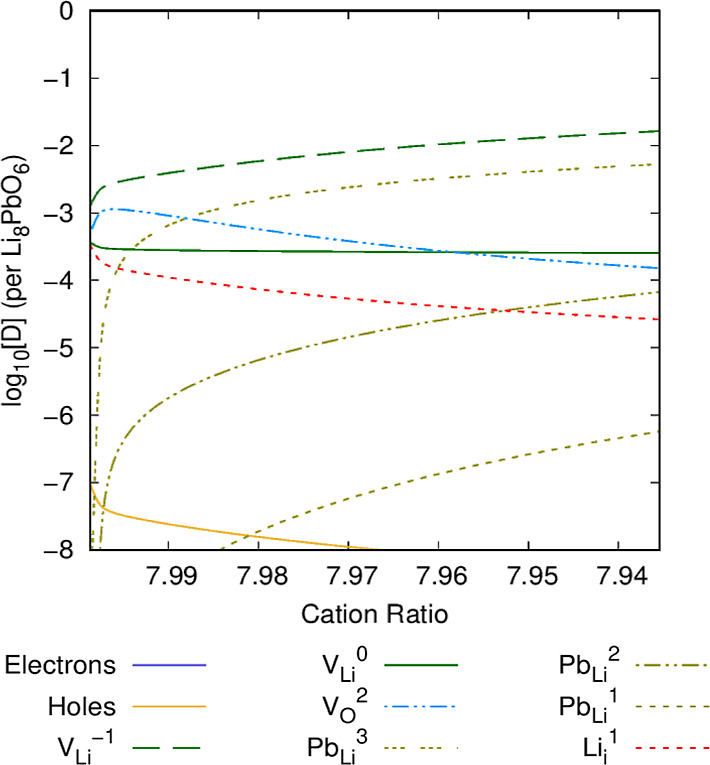
Intrinsic defect concentration in Li_8_PbO_6_ as a function of lithium burn-up. *T* = 1000 K, OPP = 0.2 atm.

**Figure 14:**
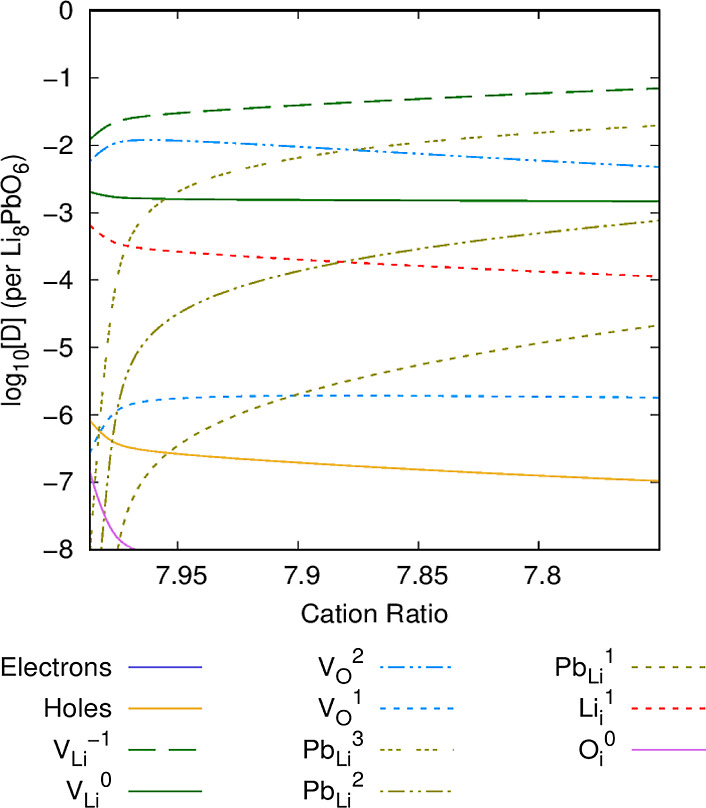
Intrinsic defect concentration in Li_8_PbO_6_ as a function of lithium burn-up. *T* = 1200 K, OPP = 0.2 atm.

The defect chemistry is expected to change throughout the operating lifetime of the ceramic as lithium is progressively used due to transmutation. Figures 12–14 show the defect chemistry of Li_8_PbO_6_ as a function of the Li/Pb ratio in the system at 800, 1000, and 1200 K, respectively. In each case, the *x*-axis extends to the point at which the chemical potential reaches the PbO_2_ saturation point. This measure is analogous to the changing defect chemistry presented throughout the temporal lifetime of Li_8_PbO_6_.

At 800 K (Figure 12), the limit at which the PbO_2_ saturation limit is reached is predicted to be at a ratio of Li/Pb of 7.991(5), meaning there is very little room for lithium burn-up before a transition to an alternative ternary phase at this temperature. Examining the defect chemistry, as expected, the lithium vacancy is the dominant defect and becomes more pronounced as lithium is depleted from the system, with Li_*i*_^1^ interstitials depleting proportionally with the increase in V_Li_^–1^. Lead begins to occupy the newly vacant V_Li_^–1^ sites as the charged Pb_Li_^3^ substitutional. All charged states of Pb_Li_ as expected increase as the proportion of the overall lead in the system increases. Interestingly, the number of oxygen vacancy V_O_^2^ defects begins to drop as Li/Pb decreases due to the overall increase in stoichiometric concentration of oxygen as lithium drops.

At 1000 K (Figure 13), the minimum possible ratio of Li/Pb is predicted to be 7.937, the limit at which a precipitate begins to form. The overall defect chemistry is broadly very similar to the 800 K case, although the primary charge-compensation mechanism is instead the V_O_^2^ vacancy defect at a high Li/Pb ratio, rather than Pb_Li_^3^.

At 1200 K (Figure 14), PbO_2_ will begin to form a precipitate at a much lower proportion of Li/Pb, at a ratio of 7.75. The concentration of lithium vacancy V_Li_^1^ is much greater at higher temperatures. The charge-compensation mechanism for V_Li_^1^ is similar to that at 1000 K, although the ratio of Li/Pb at which the compensation mechanism changes from V_O_^2^ to Pb_Li_^3^ is markedly lower (7.88 Li/Pb). At 1200 K, the overall concentration of the noncharge-compensating defects is much higher compared to lower temperatures, as is to be expected. Interestingly, the interdependence of Li_*i*_^1^ interstitials and V_Li_^0^ charge-neutral vacancies on one another is relatively insensitive to the temperature.

## Conclusions

The phase stability and intrinsic defect chemistry of Li_8_PbO_6_ were explored in this paper, with importance placed on determining the appropriateness of accounting for vibrational contributions in the phase stability and the defect chemistry by incorporating temperature contributions into the energies of the reference states for the constituent binary oxides. For high temperatures, it is shown that including these temperature effects does result in a different picture for the defect chemistry. This indicates that when considering materials operating at high temperatures, it is not appropriate to neglect the temperature-induced changes to the reservoirs as it often done.

Charged lithium vacancy defects appear to dominate the intrinsic defect chemistry for Li_8_PbO_6_ under almost every condition measured. Incorporating temperature effects appears to predominantly impact the high-temperature charge compensation mechanism for V_Li_^–1^. This may have a significant impact on the tritium migration mechanism through the crystal lattice, predominantly due to the higher predicted concentrations of oxygen vacancy defects at reactor operating temperatures. Most importantly, Li_8_PbO_6_ has been predicted to have only a stable phase close to the Li_2_O saturation limit.

Future work will examine how tritium is accommodated in the crystal and the mechanisms for diffusion through the bulk.
